# *Ascl1 (Mash1)* Knockout Perturbs Differentiation of Nonneuronal Cells in Olfactory Epithelium

**DOI:** 10.1371/journal.pone.0051737

**Published:** 2012-12-17

**Authors:** Richard C. Krolewski, Adam Packard, Woochan Jang, Hendrik Wildner, James E. Schwob

**Affiliations:** 1 Department of Anatomy and Cellular Biology, Tufts University School of Medicine, Boston, Massachusetts, United States of America; 2 Program in Cellular, Molecular, and Developmental Biology, Sackler School of Graduate Biomedical Sciences, Tufts University School of Medicine, Boston, Massachusetts, United States of America; 3 Institute of Pharmacology and Toxicology, University of Zürich, Zürich, Switzerland; Barnard College, Columbia University, United States of America

## Abstract

The embryonic olfactory epithelium (OE) generates only a very few olfactory sensory neurons when the basic helix-loop-helix transcription factor, ASCL1 (previously known as MASH1) is eliminated by gene mutation. We have closely examined the structure and composition of the OE of knockout mice and found that the absence of neurons dramatically affects the differentiation of multiple other epithelial cell types as well. The most prominent effect is observed within the two known populations of stem and progenitor cells of the epithelium. The emergence of horizontal basal cells, a multipotent progenitor population in the adult epithelium, is anomalous in the *Ascl1* knockout mice. The differentiation of globose basal cells, another multipotent progenitor population in the adult OE, is also aberrant. All of the persisting globose basal cells are marked by SOX2 expression, suggesting a prominent role for SOX2 in progenitors upstream of *Ascl1*. However, NOTCH1-expressing basal cells are absent from the knockout; since NOTCH1 signaling normally acts to suppress *Ascl1* via HES1 and drives sustentacular (Sus) cell differentiation during adult epithelial regeneration, its absence suggests reciprocity between neurogenesis and the differentiation of Sus cells. Indeed, the Sus cells of the mutant mice express a markedly lower level of HES1, strengthening that notion of reciprocity. Duct/gland development appears normal. Finally, the expression of cKIT by basal cells is also undetectable, except in those small patches where neurogenesis escapes the effects of *Ascl1* knockout and neurons are born. Thus, persistent neurogenic failure distorts the differentiation of multiple other cell types in the olfactory epithelium.

## Introduction

The main olfactory epithelium (OE), the primary sensory tissue responsible for olfaction, contains two or more stem and progenitor cell populations that establish, maintain, and reconstitute this tissue throughout the lifetime of an animal [1]–[10]. The cascade of neurogenic basic-helix-loop-helix transcription factors triggered by ASCL1 expression (also known as MASH1) and the canonical Notch signaling pathway that directly or indirectly regulates that cascade play a key role in the development and regeneration of the OE [11]–[15]. Targeted knockout animals, overexpression studies, and the changes in expression of multiple components of the pathway downstream of NOTCH following injury emphasize its importance in regulating olfactory epithelial cell fate – for example, the choice between generation of OSNs vs. other cell types [11]–[14]. In addition, the effects of removing the bHLH transcription factor ASCL1 on the neuronal progenitor population have been studied extensively [11], [15], [16]. The previous work puts ASCL1 at a crucial choice point in olfactory neurogenesis, setting in motion a cascade of transcription factors that culminates in the production of OSNs. For example, NEUROG1 and NEUROD1 are downstream of ASCL1 on the basis of timing of expression and genetic epistasis. In contrast, activation of NOTCH in multipotent progenitors upstream of ASCL1, the resulting expression of HES1, and concomitant repression of ASCL1 shifts the balance away from neurogenesis and towards a non-neuronal cell fate [12], [13], [15], [16]. As a consequence, the production of neurons by the OE is blocked almost completely by null mutations of *Ascl1* from the time of their usual appearance in the embryo onward. Because *Ascl1*knockout mice survive to the perinatal period, and because the other non-neuronal cell types are normally evident by birth, the development and differentiation of each individual cell type can be examined in response to depletion of a specific population of neuronal progenitors [3], [9], [17]–[19].

We find that blocking olfactory neurogenesis by null mutation of *Ascl1* dramatically alters the status of other differentiated cell types established and maintained by the OE. By comparison with heterozygous littermates, the *Ascl1*knockout epithelium exhibits a profound disruption in the development of horizontal basal cells (HBCs), and an alteration of gene regulatory pathways in the remaining cell types, consistent with a diversion of progenitors away from non-neuronal cell fates toward a blocked neurogenic pathway. This study highlights the interdependence of various cell types within the olfactory epithelium and documents the shifting balance of cell fate when the population of neurons and neuronal committed progenitors remains depleted.

## Materials and Methods

### Animals

Δ*Mash1-GFP* animals (referred to as *Ascl1* for the purposes of this work) have already been described [20] and were maintained on *ad libitum* rodent chow and water. All animals were housed in a heat- and humidity-controlled, AALAC-accredited vivarium operating under a 12∶12-hour light-dark cycle. Male and female heterozygous transgenic animals were mated and the morning of vaginal plug detection was taken as E0.5. Crown-rump length and Theiler staging was used to confirm embryo ages. All protocols governing the use of vertebrate animals were approved by the Committee for the Humane Use of Animals at Tufts University School of Medicine, where the animals were housed and experiments were conducted.

### Tissue Harvesting and Genotyping

Data reported herein have been compiled from the examination of multiple embryonic and perinatal time points for wild type, heterozygous and knockout animals. Four animals (2 heterozygote, 2 knockout) were examined at E12.5. Six animals (3 heterozygote, 3 knockout) were examined at E14.5. Six animals from two litters were examined at E16.5. Three animals (1 heterozygote, 2 knockout) were examined at E18.5. Fourteen animals (4 wild-type, 3 heterozygote, 7 knockout) from five litters were examined at E19.5 and PND0. For the isolation of embryonic tissue, pregnant dams were euthanized by injection of a cocktail of ketamine (37.5 mg/kg), xylazine (7.5 mg/kg) and acepromazine (1.25 mg/kg) and the uteri were removed into petri dishes containing PBS. Embryos were dissected out of the uterus and amniotic sac. Tissue samples were taken for genotyping and embryos were immersion fixed with either 4% paraformaldehyde (Fisher Scientific, Suwanee, GA) in 0.05 M sodium phosphate buffer, pH 7.2, Zamboni’s fixative [21], or Carnoy’s fixative, as indicated, for 4–6 hours. For the isolation of perinatal tissue, pups were deeply anesthetized shortly after birth with the above cocktail. Tail tissue was taken for genotyping and the pups were transcardially flushed with PBS, and perfused with one of the fixatives. The animal carcasses were decapitated and post-fixed under vacuum for 2 hours. Tissues were rinsed with PBS, cryoprotected by progression through increasing concentrations of sucrose in PBS (10% to 20% to 30% every 12 hours), and then frozen in OCT compound (Miles Inc.,Elkhart, IN). The olfactory mucosa was sectioned on a cryostat (Leica) in the coronal or sagittal plane; 12 µm sections were collected on to “Plus” slides (Fisher Scientific) and stored at −20°C for future applications. Tissue harvested for genotyping was digested in 300 µL of 50 mM NaOH for one hour at 95°C, vortexed, and 16.7 µL of 1 M Tris-HCl pH 8 was added. The samples were vortexed and spun down at 13,000 x *g* for 6 minutes. 3 µL of the DNA prep was used in separate reactions to identify a 664 bp wild type allele and a 466 bp transgenic allele (wild-type forward: GGGTTCTCCGGTCTCGTCCTACT, wild-type reverse: GCCCACCCCTGTTTGCTGAGAA, Tg forward: AAACCTCCCACACCTC CCCCTGAA, Tg reverse:ATGCCTCACCTCGACCTTCTGCTC) [20].

### Antibody Used

Please see Table 1 for a list of all antibodies used. Information of the antibodies is derived from the manufacturers’ description and our own data.

**Table 1:** Antibodies used in this study.

Primary antibody	Source and catalog number	Immunogen and preparation
Rabbit α-PGP9.5	Cedarlane/Ultraclone, RA95101	Human PGP9.5 protein purified from pathogen free human brain
Rabbit α-Hes1	Gift of T. Sudo	C-terminal peptide of Hes1 (SPSSGSSLTSDSMWRPWRN) coupled to keyhole limpet hemocyanin
Rabbit α-K18	Abcam, 52948	A synthetic peptide corresponding to residues on the C-terminus of human Cytokeratin 18
Mouse α-βIV Tubulin	Sigma, T7941. Ascites fluid ONS.1A6	Synthetic peptide corresponding to the C-terminal sequence of β-tubulin isotype IV, conjugated to BSA.
Mouse α-K14	Vector, #VP-C410	Synthetic peptide corresponding to C-terminus region of human keratin 14 (GKVVSTH EQVLRTKN) conjugated to thryoglobulin.
Rabbit α-K14	LabVision, #RB-9020-P1	A synthetic peptide of 15 amino acid residues from the C-terminus of human keratin 14.
Goat α-Sox2	Santa Cruz, sc-17320	Amino acids 277–293 of human Sox2 affinity-purified. YLPGAEVPEPAAPSRLH.
Rabbit α-Notch1	Abcam, ab27526	Synthetic peptide (AASGHLGRSFLSGEPSQADV) from C-terminal of human notch-1.
Rabbit α-Notch1	Cell Signaling Technology, #3608	Synthetic peptide corresponding to residues surrounding Pro2439 of human Notch1.
Mouse α-Ki67	BD-Biosciences, 556003 (clone B56)	22 aa Ki-67 repeat motif (APKEKAQPLEDLASFQELSQ)
Rat α-NCAM	Abcam, ab19782 (H28-123)	Glycoprotein fraction from neonatal mouse brain
Mouse α-TuJ1	Covance, MMS-435P	Microtubules derived from rat brain.
Rabbit α-Phospho-Histone H3 (Ser10)	Millipore, 06-570	Linear peptide corresponding to human Histone H3 at Ser10.
Rabbit α-PCNA	Abcam, ab2426-1	Synthetic peptide: DMGHLKYYLAPKIEDEEGS, corresponding to C terminal amino acids 243–261 of Human PCNA.
Rabbit α-cKit	Cell Signaling Technology, #3074	Synthetic peptide corresponding to the residues surrounding Tyr703 of human c-Kit.
Mouse α-SUS4	Schwob Lab	Rat OE
Rabbit α-Ezrin	Gift of M. Berryman	Ezrin from human placenta
Rabbit α-REEP6	Abcam, ab104097	Synthetic peptide conjugated to KLH, corresponding to C-terminal aa (164–194) of human REEP6

The PGP9.5 antiserum recognizes a single 27 kDa band on Western blots of mouse brain and cross-reacts with all mammalian species tested. [22]. The sub-cellular labeling for PGP9.5 is cytoplasmic and restricted to neural populations in mice, including within the vomeronasal organ and olfactory epithelium [23], [24].

The anti-HES1 antibody recognizes *in vitro* -translated Hes-1 protein and the native Hes-1 antigen in the nuclei of transfected NIH-3T3 cells. In mice lacking Hes-1, no expression was found in the stomach and intestinal villi of *Hes1* ^−/−^ embryos [25].

The anti-K18 antibody detects a band of approximately 48 kDa on WB that corresponds in molecular weight to K18 (Abcam datasheet).

The anti-βIV tubulin antibody recognizes an epitope located in the C-terminal sequence of β-tubulin isotype IV. No reactivity with other tubulin isotypes is observed.

The mouse anti-K14 monoclonal antibody VP-C410 labels basal cells in skin, selectively, with a cytoplasmic distribution (Vector Laboratories Datasheet VP-C410). The staining pattern with VP-C410 is completely equivalent to that observed with another anti-K14 monoclonal, RGE-53, which labels a single spot in 2-D IEF-SDS-PAGE western blot with a molecular weight and isoelectric point consistent with K14 [19]. The choice of VP-C410 was predicated on the retention of antigenicity despite formaldehyde fixation.

The rabbit anti-K14 monoclonal antibody produces staining identical to that generated by the mouse anti-K14 described above. Specifically, it stains A431 cells and skin or squamous cell carcinoma (Lab vision data sheet).

The goat anti-Sox2 antibody detects a single band of 34 kDa in western blot of mouse and human embryonic stem cell lysates (clone Y-17: datasheet # sc-17320) and antibody staining on embryonic tissue from Mash1 knockout olfactory epithelium replicates the published Sox2 mRNA pattern by ISH [26]. Conditional deletion of Sox2 extinguishes immunolabeling in mouse cortex by the antibody [27].

The rabbit anti-Notch1 antibody (ab27526) recognizes NOTCH-1 in western blots of breast carcinoma tissue (Abcam datasheet). The rabbit anti-Notch1 antibody (#3608) recognizes both the full-length (∼300 kDa) and the cleaved transmembrane/intracellular region NTM (∼120 kDa) on WB of HBP-ALL, MOLT4, and TALL-1 cell lysates. The staining patterns generated by these two antibodies in olfactory epithelium are identical.

The Ki-67 rabbit antiserum reacts with 345/395 kDa doublet on western blot corresponding to Ki-67 Ag; B56 binding to cells is blocked by clone MIB 1, the canonical anti-Ki67 antibody.

The rat anti-NCAM antibody recognizes a triplet of glycoproteins at the neural cell surface that is identified as neural BSP2, which is a synonym for NCAM (Abcam datasheet).

Tuj1 is a mouse monoclonal antibody that is highly reactive to neuron specific Class III β-tubulin (III) and does not label β-tubulin found in glial cells.

The rabbit anti-phospho-histone 3 was evaluated by western blot of Colcemid treated HeLa acid extract, such that 0.5–1 µg/ml of this antibody detected Histone H3 on 10 µg of the extract (Millipore Datasheet).

The rabbit anti-PCNA antibody detects a band of approximately 29 kDa (predicted molecular weight of PCNA), which can be blocked with PCNA peptide (ab2427) (Abcam datasheet).

The rabbit anti-c-Kit antibody detects bands at 120 and 145 kDa corresponding to cKit and not other receptor tyrosine kinases on western blot of NCI-H526 cells. ICC of NCI-H526 shows staining not present in control Jurkat cells.

The following secondary antibodies were used, AlexaFluor488-donkey anti-goat IgG, AlexaFluor488-Donkey anti-mouse IgG, AlexaFluor488-Donkey anti-rabbit IgG, AlexaFluor594-Donkey anti-goat IgG, AlexaFluor594-Donkey anti-mouse IgG (all from Invitrogen/Molecular Probes) each used at a dilution of 1∶250. Additionally, AlexaFluor488-conjugated streptavidin and AlexaFluor594-conjugated streptavidin (Invitrogen/Molecular Probes) were used at dilutions of 1∶250 for conventional immunofluorescence, but at a dilution of 1∶1250 for tyramide signal amplification (TSA). The remaining secondary antibodies were used at a dilution of 1∶50 and were purchased from Jackson Immunoresearch: biotin-conjugated donkey anti-goat IgG, biotin-conjugated donkey anti-mouse IgG, biotin-conjugated donkey anti-rabbit IgG, FITC-conjugated donkey anti-goat IgG, FITC-conjugated donkey anti-rabbit IgG, AMCA-conjugated donkey anti-mouse IgG, Texas Red-conjugated donkey anti-goat IgG.

### Immunohistochemistry

Standard laboratory protocols were used for immunohistochemical detection of the expression pattern of individual proteins in normal vs. mutant OE [23] (Table 1). Adequate labeling with a number of the antibodies requires a set of treatments on the sections prior to incubation with the antibodies. Briefly, frozen sections were rinsed in PBS for 5 minutes to remove the OCT and then boiled in 0.01 M citric acid buffer (pH6.0) for 10 minutes using a standard kitchen food steamer. After cooling, sections were rinsed with PBS briefly before incubating with block (10% serum +5% Non fat dry milk +4% BSA +0.01% TritonX-100) for 30 minutes at room temperature. The analyses conducted here depended on a number of double and triple-immunohistochemical staining approaches. In all cases, the sections were incubated with primary antibodies overnight at 4°C. The visualization of bound primary antibodies varied depending on the nature of the staining. Methods included biotinylated secondary antibody followed by avidin-bHRP conjugate (Elite ABC Kit, Vector Laboratory, Burlingame, CA) and 3,3′-Diaminobenzidine (DAB) as chromagen, or in the case of co-localization studies with one of several fluorescently-conjugated secondary antibodies. On occasion, tyramide signal amplification was used to enhance a weak signal or permit staining with two antibodies from the same species, and used according to kit instructions (Perkin-Elmer).

### Photography

Sections were imaged with a Spot RT2 color digital camera mounted on a Nikon E800 microscope. Image preparation, assembly and analysis were performed in Photoshop CS2 and CS3. Except for Figure 8, only balance, contrast and evenness of the illumination were altered. In Figure 8, the low power images were filtered with an unsharp mask to enhance contrast (pixel = 2, amount = 66, intensity = 0).

### Image Analysis

For all pixel intensity measurements, original raw TIFF files were opened in ImageJ (NIH). For quantitation of HES1 immunoreactivity, an image of the nasal mucosa was acquired such that both olfactory and respiratory epithelium were visible and enough HES1-immunoreactive cells were present such that pixel intensity could be measured from 50 single pixel spots per section in the OE and 50 single pixel spots per section from the RE. A ratio of pixel intensity was calculated for each genotype and developmental time point by dividing the average pixel intensity of 50 spots in the olfactory epithelium by the average pixel intensity for 50 spots in the respiratory epithelium on that same section.

### HBC Counts and Length of Epithelium at the OE-RE Boundary Covered by HBCs

The density of HBCs in the OE was determined in both knockout and wild type mice harvested at E19.5 (n = 3 for each). The OE was divided into two territories for purposes of the initial counts: the junctional region next to the boundary between olfactory and respiratory epithelium and the rest of the OE. In order to establish that division as objectively as possible, the distance from the boundary to the top of the vault of the dorsal recess of the nasal cavity was measured along both the septum and the lateral wall, and the junctional region was defined as that portion that was 20% and less of that distance, as this was the average distance occupied by the banc of contiguous HBCs in the wild-type epithelium. Matched sections were counted through the anteroposterior extent of the OE for each case. Measurements of HBC cell size were made in the knockout and wild type animals. The values obtained were 9.45±0.55 µm (mean ± S.E.M.) and 9.05±0.46 µm. Raw profile counts were corrected using the Abercrombie formula and the measurements of cell size [28]. In addition, the distance along the basal lamina of the OE from the olfactory-respiratory border to which the contiguous band of HBCs extends was also measured in the knock-out epithelium.

## Results

The *Ascl1* knockout line used in this work has not previously been analyzed with respect to the cellular composition and differentiation of the OE [20]. By comparison with the published descriptions of another Ascl1 knockout strain, our results are highly analogous but extend well beyond the previous analyses [15], [16], [29]. As described before, the OE is thinner in the homozygous mutants, and the OE is nearly, but not completely, aneuronal by the later stages of embryogenesis (data not shown). Olfactory sensory neurons were identified by labeling with monoclonal antibody TuJ1 (to stain neuron-specific tubulin) and anti-PGP9.5 from the earliest stages in their differentiation. Both markers demonstrate the dramatic reduction of neurons in the OE of knockout mice from E12.5 onward, but they also mark a small, but consistent, subset of neurons situated in the part of the mutant OE adjacent to respiratory epithelium, as was reported previously [16] as well as in a few other epithelial locations as described below.

The current findings provide novel insights into the epithelium-wide regulation of stem cell function by assessing the differentiation of other cell types and progenitor populations in a setting where neurogenic failure is ongoing.

### Sustentacular Cell Differentiation in the Olfactory Epithelium

**Figure 1:**
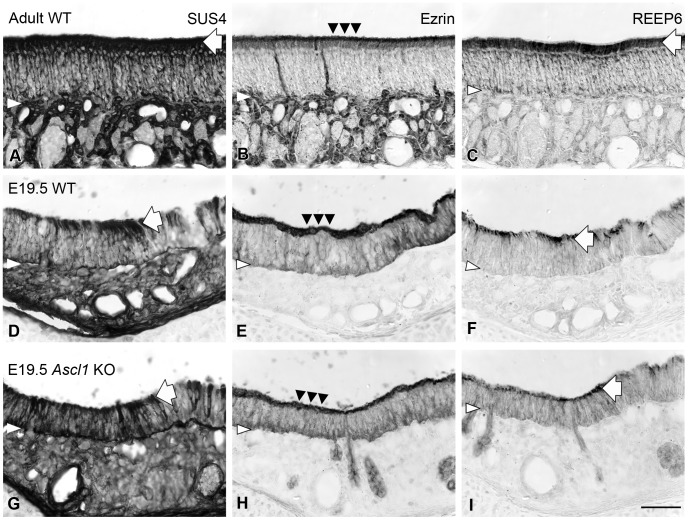
Sus cells differentiate normally in developing OE of Ascl1 KO mice. Sustentacular (Sus) cells are marked in adult wild-type mice (**A–C**) and in wild-type embryos (**WT;** **D–F**) and knockout (***Ascl1*** **KO;** **G–I**) littermates at E19.5 by staining with **SUS4** (**A, D, G**), anti-**ezrin** (**B, E, H**) and anti-**REEP6** (**C, F, I**), as shown here on three consecutive sections from each animal. Prominent cytoplasmic labeling of both **SUS4** and anti-**REEP6** at the apical processes of Sus cells (*large white arrows*) is comparable between wild-type and knockout mice. Anti-**ezrin** heavily labels the microvilli at the apical surface of the Sus cells (*black triangles*) as well as Bowman’s duct/gland cells in the adult (**B**) and the staining pattern is consistent in wild-type (**E**) and knockout (**H**) mouse embryos. *White arrowheads* indicate the basal lamina. Scale bar in **I** is 50 µm, and also applies to **A–H**.

Previous reports suggest that levels of *Hes1* mRNA are markedly diminished in the olfactory epithelium of Ascl1 knockout mice at E12.5 [12]. Later in development sustentacular (Sus) cells exhibit an altered molecular phenotype [15]. Nonetheless, we see no evidence of aberrant development of Sus cells with respect to morphology and immunotype by direct visual examination using DIC microscopy and immunohistochemical staining with a number of Sus cell-specific marker antibodies. Microvilli-capped cells line the apical surface of the olfactory epithelium of both heterozygote and knockout at E14.5, suggesting that the differentiation of Sus cells is progressing at a normal pace (data not shown). By E19.5, all of the markers that are known, or here are shown, to label Sus cells produce equivalent staining in both wild-type and knockout mice (Fig. 1). For example, SUS4 produces intense somatic staining of Sus cells by SUS4 in both wild-type (Fig. 1D) and knockout (Fig. 1G) epithelium, even though the staining on the foot processes is weaker in either genotype compared to that in wild-type adults (Fig. 1A). Likewise, the expression patterns of ezrin in the apical microvilli and of REEP6 in the supranuclear region of Sus cells in the knockout mice (Fig. 1H, I, respectively) are similar to wild-type littermates (Fig. 1E, F, respectively). Several other antibodies that label Sus cells in the fully adult OE stained neither the epithelium of knockout nor wild-type mice at pre- and neonatal ages, including LKB-1, ZO-1, and E-cad (negative data not shown).

**Figure 2:**
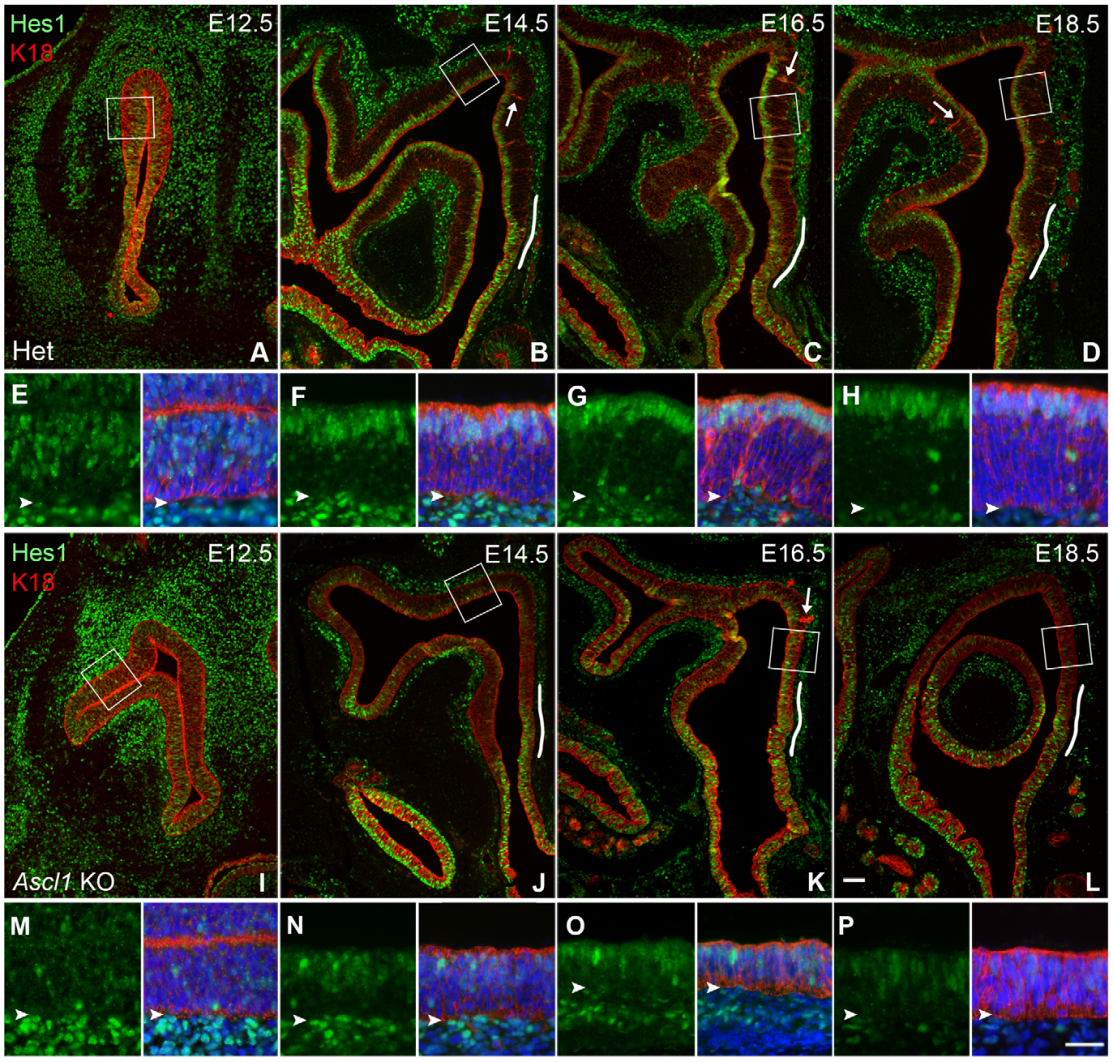
Sus cells differentiate on schedule, but lack robust HES1 expression in the *Ascl1* KO OE. Sections of nasal epithelium harvested at various embryonic time points were stained with **K18** (to label differentiating Sus cells), **HES1**, and **Hoechst 33258** (to label nuclei). Boxed areas in **A**–**D**, **I**–**L** are shown at higher magnification beneath each image as the green channel (*left image* for **HES1** staining) and three channel merged images (*right image*). White lines overlaying the basal lamina in **B**–**D**, **J**–**L** highlight the transitional area from respiratory epithelium situated ventrally to olfactory epithelium situated dorsally. Arrows in **B–D, K, L** show developing duct/gland units. Arrowheads in high magnification panels (**E–H** and **M–P**) indicate basal lamina. In heterozygote (**HET**), **HES1**-expressing differentiating sus cells coalesce to form a tight apical layer by E14.5 and maintain this pattern throughout subsequent development, with the exception of rare scattered cells deep in the OE. In contrast, in *Ascl1* knockout (***Ascl1*** **KO**) some apical nuclei lack detectable labeling with **HES1** antibody. This layer of differentiated sus cells is less compact and the relative overall reduction in **HES1** expression is indicated by comparison with respiratory epithelium; there is a sharper drop-off of RE to OE in the ***Ascl1*** **KO**. Arrowheads in insets indicate basal lamina. Scale bar in **L** (50 µm) applies to **A**–**D**, **I–L**; scale bar in **P** (20 µm) applies to **E–H, M–P**.

Despite the equivalence of Sus cell differentiation between the knockout and wild-type epithelium, we extended the previous descriptions of aberrant *Hes1* gene expression [16] by investigation of HES1 protein expression in the absence of *Ascl1* by HES1 immunohistochemical analysis [30] from E12.5 onward through the immediate postnatal period, using an anti-K18 antibody to mark Sus cells and duct/gland cells (Fig. 2). As something of a surprise given previous reports [16], HES1 protein is detectable in at least some apically located cells of the developing olfactory epithelium of the knockout mice, which may reflect a relative insensitivity and lack of spatial resolution of detection of *Hes1* mRNA by *in situ* hybridization vs. antibody labeling of HES1 protein (Fig. 2). However, the intensity of HES1 labeling in the knockout OE is decidedly less overall as compared with wild type or heterozygous mice. Moreover, the layer of HES1 (+) cells is very disorganized in the mutant epithelium.

In the heterozygous and wild-type mice, HES1(+) Sus cells form a compact layer superficial to the neuronal strata (as soon as it begins to emerge), while in the knockout mice HES1(+) cells are poorly laminated at all ages examined (Fig. 2). Thus, at E12.5 the band of HES1(+) Sus cells is concentrated in the upper region of the epithelium in the heterozygotes (Fig. 2A, E) or wild-type mice (data not shown). In contrast, no such band of labeling is seen in the knockout OE, which also appears thinner and more disorganized. Strongly HES1(+) epithelial cells are exceptionally rare in the knockout at this point (Fig. 2I, M) and remain rare at all subsequent ages, by comparison with the heterozygote. At E14.5, the difference between the heterozygote and knockout with regard to the stratification of the HES1(+) Sus cells becomes more striking as the Sus layer becomes compact; the difference in overall thickness of the OE is also substantial between heterozygote and knockout (Fig. 2B, F, J, N). Some HES1(+) nuclei are detectable in the knockout OE at this stage and are found both at the apex as well as deeper in the OE. However the magnitude of the difference between heterozygote and knockout is made particularly manifest by reference to the staining of the respiratory epithelium (cf., Figs. 2B and 1J). There is a sharp fall-off in HES1 immunostaining at the olfactory-respiratory boundary in the knockout but not in the heterozygote (the junctional region is marked by the white line superimposed on the lamina propria in Fig. 2). Over the next several days to E18.5, the pattern of HES1/K18 staining remains largely the same (Fig. 2). In the heterozygote, the OE progressively thickens and the layer of HES1(+)/K18(+) Sus cells grows tighter. In addition, the HES1 antibody now labels other OE cell types, namely duct/gland cells and a population of globose basal cells that are K18(−). In the knockout, the layer of Sus cells remains loosely arranged at this time, and HES1(+)/K18(−) cells are found throughout the height of the OE.

With regard to the level of HES1 expression in the knockout OE, some apically located K18(+) cells, which are presumptive Sus cells, lack detectable HES1 immunoreactivity, and the Hes1 labeling is lighter in the remaining cells as compared to the heterozygote. The reduced expression is evident by two measures: 1) by comparing the relative fall-off in the intensity of HES1 labeling along the traverse from RE to OE, which is more pronounced in the knockout than in the heterozygote (Fig. 2); 2) by measuring the pixel intensity of HES1 staining on a cell-by-cell basis. Measures of staining intensity, even when limited to those cells in which HES1 expression is detectable (i.e., neglecting the K18(+) cells that lack staining) demonstrate that the HES1 labeling in the knockout is less intense on a per-cell basis compared to heterozygote, when HES1 signal intensity is normalized to the expression level in the lamina propria and respiratory epithelium (RE) (Table 2). Normalizing to RE, a tissue in which ASCL1 is normally absent, minimizes technical causes of variation in staining between multiple samples.

At all time points examined, the ratio of OE to RE HES1 pixel intensity was greater in the heterozygote compared to knockout (Table 2). At E12.5 there is little or no difference in the pixel intensity ratio (heterozygote – knockout = 0.039), but by E14.5 onward to birth, there is a larger difference between the pixel intensity ratios of the heterozygote and knockout (0.595, 0.169, and 0.315 for E14.5, E16.5, and E18.5, respectively).

**Table 2:** The labeling intensity of Hes1 is reduced in Ascl1 knockout compared to heterozygote.

Embryonic Age	Genotype	Region	Avg Pixel Intensity	Standard Deviation	Range	OE/RE Pixel Intensity Ratio
**E12.5**	Het	OE	76.3	17.10	35–116	**0.759**
		RE	100.48	21.93	48–154	
	KO	OE	57.98	16.36	33–122	**0.720**
		RE	80.54	19.99	37–140	
**E14.5**	Het	OE	78.26	24.40	35–163	**0.910**
		RE	85.96	29.95	38–215	
	KO	OE	23.4	11.75	9–62	**0.315**
		RE	74.28	26.70	28–135	
**E16.5**	Het	OE	144.47	29.55	84–222	**1.005**
		RE	143.69	33.53	74–227	
	KO	OE	98.43	28.52	56–200	**0.836**
		RE	117.78	29.31	79–234	
**E18.5**	Het	OE	50.98	20.16	17–146	**0.607**
		RE	83.98	34.12	28–181	
	KO	OE	32.02	10.03	11–64	**0.292**
		RE	109.64	40.29	41–253	

Multiple sections from various ages were stained for Hes1 and imaged. The intensity of nuclear labeling was sampled from 50 consecutive nuclei and pixel intensity for 50 single-pixel intensity readings were measured for Hes1(+) consecutive nuclei in the olfactory domain and the respiratory domain using ImageJ (in other words, unstained cells were not measured). A ratio of intensity values was calculated for olfactory epithelium to respiratory epithelium for each genotype at each embryonic time point described. At all time points examined the calculated OE/RE intensity ratio is less for **Ascl1 knockout** **mice** than **heterozygotes**.

### Delayed Horizontal Basal Cell Development

HBCs have recently been shown to function as a reserve population of multipotent progenitor cells in the adult olfactory epithelium that is activated to participate in reconstitution of the OE following injury [5]. Published work shows that the K5/K14(+) basal cells of the RE emerge before birth well in advance of the HBCs of the OE [19]. For example even at late prenatal stages in rat and mouse OE, HBCs are evident only near the boundary with RE, and the population gradually expands and forms a monolayer apposed to the basal lamina throughout the OE by two weeks of age [19], [31]. The sequence and timing of the establishment of the HBCs is very similar in normal mice in heterozygous animals as compared to wild type or normal mice [31]. However, we find that the emergence and accumulation of K14-marked HBCs is significantly diminished, but not completely eliminated, during development as a result of the *Ascl1* knockout.

**Figure 3:**
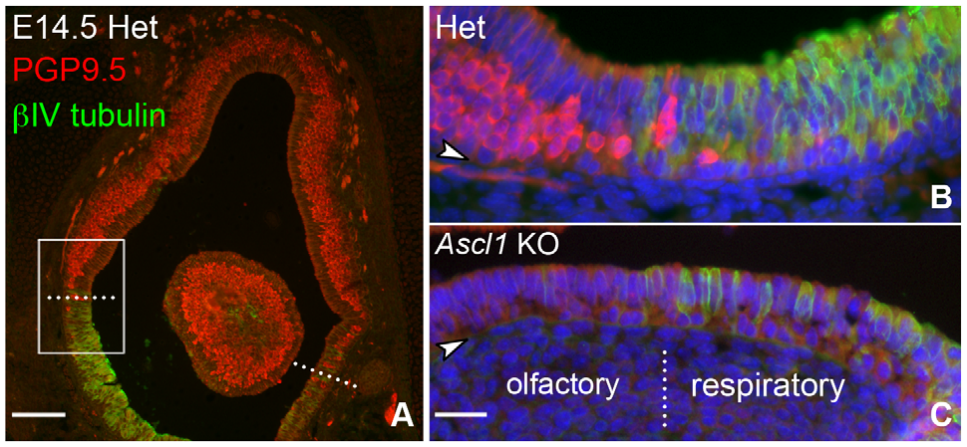
βIV TUBULIN strongly labels respiratory epithelium. Sections of nasal epithelium were harvested from **E14.5 HET** and ***Ascl1*** **KO** animals and stained for **PGP9.5**, a marker of neuronal differentiation, and **βIV TUBULIN**, which strongly labels respiratory epithelium. Dotted lines identify the olfactory/respiratory border. Boxed region in **A** is shown at higher magnification in **B**. Note that the **βIV TUBULIN** labeling is strictly limited to ventral epithelium and has minimal overlap with the **PGP9.5**(+) olfactory epithelium, substantiating its usefulness as a respiratory epithelial marker. **C.** An image of the olfactory/respiratory border in age-matched *Ascl1* KO littermates, showing the expected lack of neurons (lack of **PGP9.5** labeling) in the olfactory area and a region of **βIV TUBULIN(+)** epithelium, which marks the respiratory epithelium in these animals as well. Scale bar in **A** is 100 µm. Scale bar in **C** panel is 25 µm and applies to **B** and **C**.

In light of the patterning of HBCs in the developing OE, the assessment of HBC differentiation as a consequence of *Ascl1* null mutation must also take into account the topography of the nasal epithelium. In this case, we have used complementary labeling with PGP9.5 to mark neurons of the OE and/or βIV TUBULIN to label the ciliated brush border and cells of the RE to define the OE-RE boundary between them. In the heterozygote and in the knockout, βIV TUBULIN clearly delimits the territory of the RE (Fig. 3).

**Figure 4:**
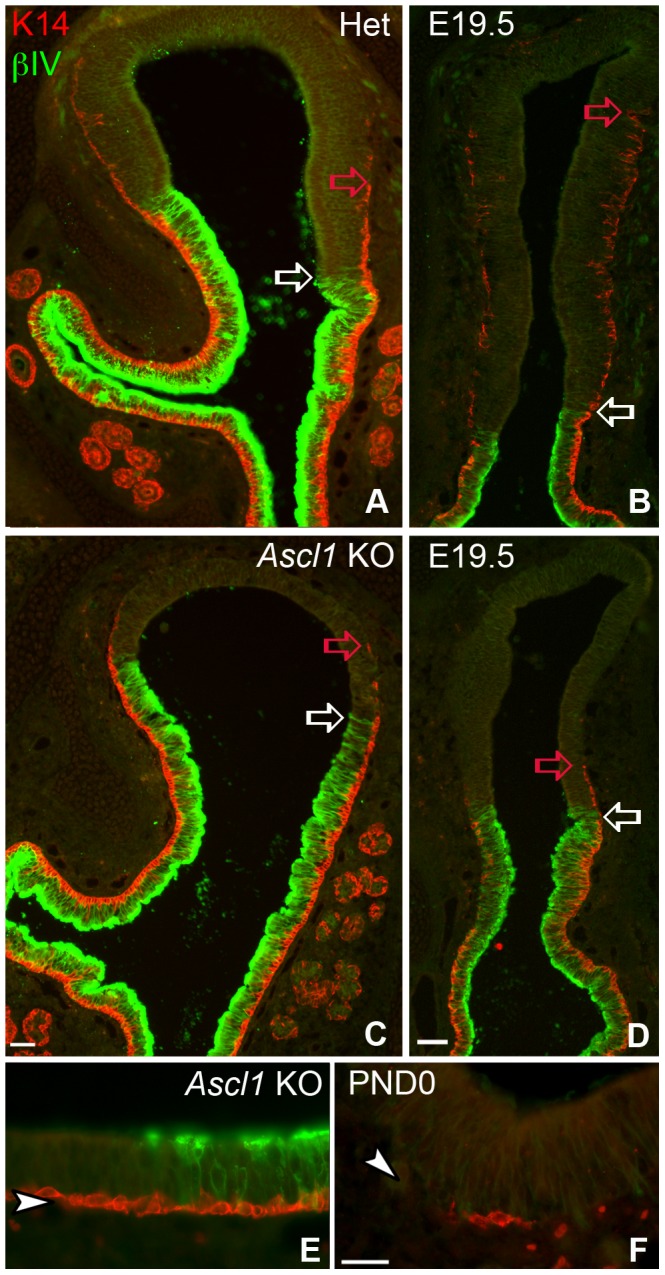
Horizontal Basal Cells are largely absent from *Ascl1* KO olfactory epithelium at E19.5. Sections of nasal epithelium at E19.5 were stained for **βIV TUBULIN** and **K14**. **K14** marks horizontal basal cells (HBCs) and **βIV TUBULIN** marks respiratory epithelium (**A–D**). Open **white arrows** designate boundary between respiratory epithelium (RE) and olfactory (OE). Open **red arrows** designate the limits of the dorsal extent of **K14** staining in OE. In **HET**, the extent differs between rostral (**A**) and caudal (**B**). (**A**)**,** (**C**) At rostral levels, **K14(+)** HBCs extend a number of cell diameters beyond the respiratory/olfactory boundary into the olfactory epithelium. At rostral levels of both **HET** and ***Ascl1*** **KO K14**(+) HBCs extend into the OE to a roughly comparable extent (**red arrows** in **A** and **C**). (**B**)**,** (**D**) In contrast, **K14** immunoreactivity at caudal levels of the heterozygotes (**HET**) is extends much further into the dorsal septum, as compared to ***Ascl1*** **KO**, where **K14** immunoreactivity (**red arrows**) is rarely found more than a few cell diameters beyond the RE/OE boundary (**white arrows**). Sections of ***Ascl1*** **KO** OE at **PND0** stained for these same two proteins indicate that **K14(+)** HBCs are found both at the border with respiratory epithelium (**E**), as well as far dorsally in the OE (**F**), indicating that the emergence of HBCs is not prevented in areas away from the OE-RE transition zone. Scale bar in **D** (50 µm) applies to **A**–**D**. Scale bar in **F** (25 µm) applies to **E** and **F**.

**Figure 5:**
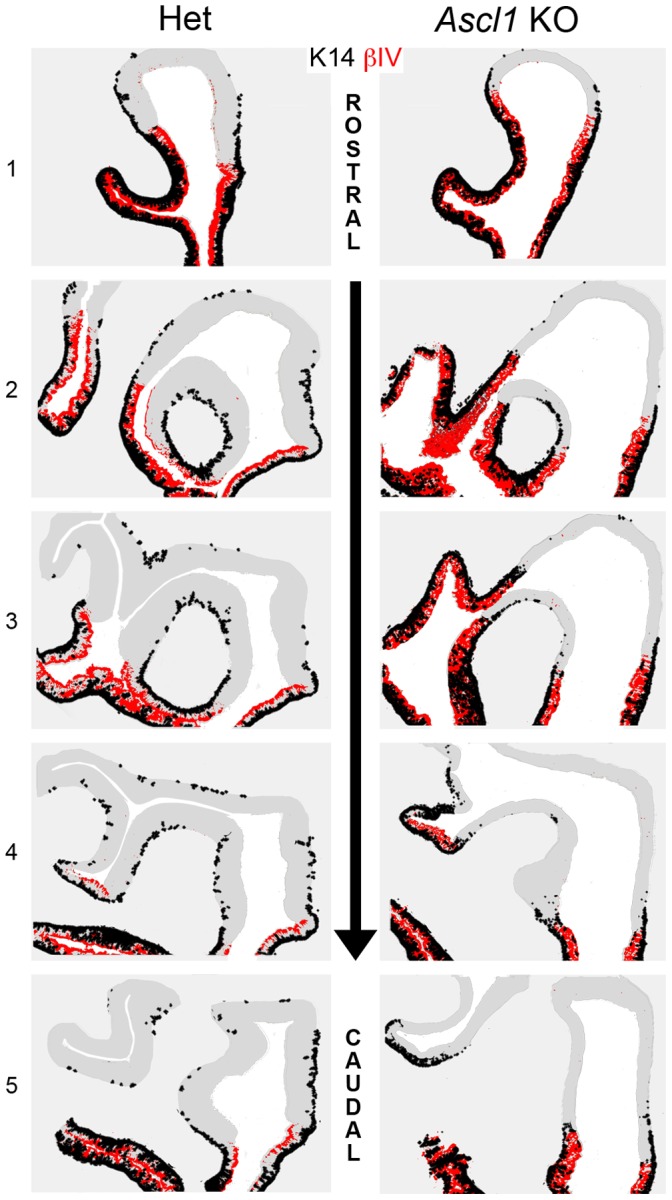
HBC Emergence is aberrant but not aborted completely as a consequence of *Ascl1* KO. Matched sections from E19.5 **HET** and **Ascl1 KO** nasal epithelium were stained for **K14** to mark HBCs and **βIV TUBULIN** to mark respiratory epithelium. **K14** staining was highlighted and cartooned in black and **βIV TUBULIN** was highlighted and cartooned in red. Representative sections were arranged from **Rostral** to **Caudal** levels (*top* to *bottom*, matched levels numbered **1** to **5**). Coincident black and red indicate regions of respiratory epithelium that contain a dense population of K14 (+) basal cells, while areas of black only represent the emergence of **K14**(+) HBCs in olfactory epithelium. In the **HET** (*left side*) HBCs are relatively abundant in the caudal olfactory areas, while few if any are seen in comparable regions of the OE in the ***Ascl1*** **KO** (*right side*).

In the wild-type mice and heterozygotes, K14(+) cells are largely, but not completely confined to the βIV(+) areas from E14.5 to E18.5; at these ages, the contiguous band of K14(+) HBCs and βIV(+) respiratory epithelial cells are coextensive and stop abruptly very near the RE/OE border. K14(+) cells are not seen more than a few cell diameters beyond the limit of the βIV(+) RE during this period, with the exception of rare, weakly K14(+) cells that are scattered in the OE and are often situated well superficial to the basal lamina. At E19.5, K14(+) cells become more prominent and are present in larger numbers, extending as a continuous layer to a greater distance from the OE/RE boundary and are more frequent, though still scattered, throughout the expanse of the OE. A relationship seems to exist between the extent to which HBCs are evident and the anterior-posterior location within the epithelium (Fig. 4). At both rostral and caudal levels of the OE, the continuous band of K14(+) basal cells in the RE crosses the boundary into the OE, but rostrally the HBCs extend further dorsally – filling much of the dorsal recess of the OE – than at more caudal levels (*cf.* the relative positions of the red arrows, Fig. 4A, B). With respect to the emerging HBCs that are dispersed across the remainder of the OE, isolated K14(+) HBCs are found scattered across the dorsal expanse of the OE at all rostrocaudal levels (Fig. 5). Thus, HBCs seem to emerge perinatally via two different patterns – as a continuous population extending dorsalward from the RE-OE boundary, and as individual, scattered cells throughout the OE without reference to that boundary.

In the knockout mice, the distribution of K14(+) cells in the mutant OE is starkly different at E19.5 and PND0 with respect to both patterns. First, HBCs do extend into the OE from the OE/RE boundary in the knockout, although the distance is less than in the heterozygote at all levels along the anteroposterior axis of the tissue (red arrows, Fig. 4A vs. C, Fig. 4B vs. D). On average, the band of confluent HBCs in the knockouts extends to only 80% of the distance covered by that band in wild-type mice. Second, there are many fewer K14(+) cells in dorsal regions of OE at a distance from the boundary (Fig. 5).

In order to document the difference in emergence of HBCs between the knockout and the wild-type OE, HBC profiles were counted separately in the junctional region of the OE near its boundary with the RE and in the rest of the OE (i.e., at a distance from that boundary) and corrected for size. The division of the OE into the junctional vs. non-junctional regions was based on the distance between the OE-RE boundary and the dorsum of the nasal vault and was placed at 20% of that distance, because that corresponds to the limit of the confluent band of HBCs that extends into the OE from the RE in the wild-type. The corrected counts for the junctional region were 3.92±0.41 HBCs/100 µm vs. 5.17±0.94 for the knockout vs. wild-type mice, respectively (t = 1.523; p (1-tail) = 0.134, n = 3) – less in the knockout but not significantly different. In contrast, the corrected counts in the rest of the OE were statistically fewer in knockout as compared to wild-type at 0.06±0.02 vs.1.42±0.45, respectively (t = 4.853; p (1-tail) = 0.020, n = 3).

These data suggest that the formation of HBCs in the knockout OE is aberrant, and perhaps delayed, but not prevented, since some K14(+) basal cells are seen near and far from the OE/RE boundary (Figs. 4C–F, 5).

### Normal Duct/Gland Development

**Figure 6:**
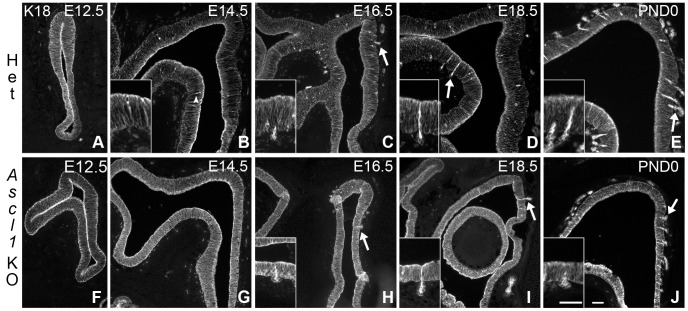
Bowman’s duct and gland development appear normal in the *Ascl1* KO OE. OE sections harvested at various ages were stained for **K18** to mark sustentacular cells and developing Bowman’s duct and gland units. In sections harvested from **E16.5** and later, examples of developing duct/gland units and gland acini are indicated by **arrows** and shown at higher magnification in insets. At **E12.5** and **E14.5**, ducts are not yet distinct. However, occasional intensely stained cells that are indicated by **arrowheads** extend toward the basal lamina, and are presumably developing ducts (**B)**. By **E16.5**, epithelial spanning ducts in the lamina propria are seen in both **HET** and **Ascl1 KO** (arrows in **C**, **D**, **H, I, J**). Insets show the duct/gland structures identified by **arrows**. Scale bar in **J** (50 µm) applies to **A–J**. Scale bar in the inset of panel **J** (25 µm) applies to all insets.

In contrast to the altered pattern of differentiation of HBCs, the timing of development of Bowman’s glands and ducts is not detectably different in the knockout mice as compared to heterozygotes. However, the architecture of the duct/gland unit in the knockout epithelium does appear more disorganized than in the heterozygotes (Fig. 6). Nascent duct/gland structures are identified by a combination of staining with anti-K18 and their characteristic morphology. With regard to the latter, ducts (which develop first) can be distinguished from sus cells by their arrangement into chains of spindle-shaped K18(+) cells whose nuclei are oriented perpendicular to the basal lamina and are found deep to the apical layer of sus cell nuclei. More mature duct/gland units are easily identified by the clusters of K18(+) cells that extend into the lamina propria and form acini. Neither heterozygous nor knockout OE has identifiable K18+ duct gland structures at E12.5. At E14.5, occasional epithelial-spanning K18(+) ducts can be seen in the OE of the heterozygotes vs knockout mice. By E16.5, the ducts frequently penetrate the basal lamina and terminate in small round glandular acini in both the heterozygotes and knockout mice, which fits with previously published light microscopic and electron microscopic descriptions of their development [2], [3]. Over the next few days, the number of duct/gland units increase in both heterozygous and knockout OE without any substantial differences in number between the two, when the epithelium is sampled and assayed through its anteroposterior extent (data not shown).

Although anti-K18 labeling in knockout littermates allows duct and gland cells to be identified in the mutant OE, nonetheless, the duct/gland units appear poorly organized by comparison with the epithelium of the heterozygotes. The strands of K18(+) duct cells appear poorly aligned and are shorter (in keeping with the thinner OE in the knockout). The glands also have abnormal morphology. The acini do not extend as deeply into the lamina propria of the knockout and the glands appear smaller and irregular in shape.

### Alterations in the Population of GBCs as a Consequence of Ascl1 Knockout

The remaining major epithelial cell type to consider is the population of GBCs. A definitive population of GBCs can be said to have emerged during the normal development of the OE when proliferating neuronal progenitors complete their translocation from an apical position (characteristic of the olfactory pit stage in the development of the OE from the olfactory placode) to a basal location. The population of GBCs has been subdivided previously on the basis of basic helix-loop-helix expression [11]–[13], [16], [32]. Additional subsets of GBCs can be identified by other markers, in particular, by the expression of NOTCH1 on their surface, by c-KIT, and by the expression of the transcription factors SOX2 and PAX6 [14], [23]; Schwob, unpublished data and below).

**Figure 7:**
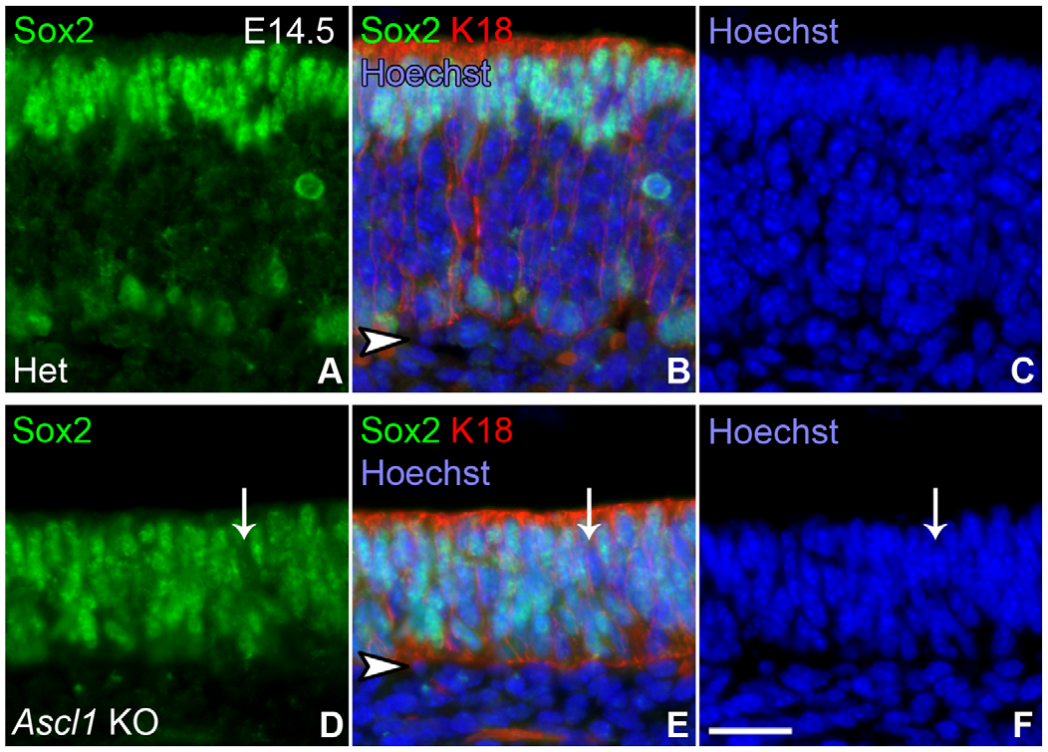
SOX2 is expressed by the overwhelming majority of the cells in the *Ascl1* KO epithelium. OE sections harvested from **E14.5** animals were stained for **SOX2**, **K18** and **Hoechst 33258**. (**A**), (**D**) Single channel green images of SOX2 staining. (**B**)**,** (**E**) Merged images of **SOX2** (green), **K18** (red), and **Hoechst 33258** (blue, to label nuclei). (**C**)**,** (**F**) Blue-only images showing labeled nuclei. (**A–C**) In **HET** epithelium anti-**SOX2** labels two major populations, apical Sus cells (identified by co-labeling with K18), and GBCs. (**D–F**) In ***Ascl1*** **KO** epithelium anti-**SOX2** stains cell nuclei throughout the entire apical to basal extent of the epithelium with only a very few exceptions (e.g., **white arrow**). **Arrowheads** indicate basal lamina. **Scale bar** in **H** is 25 µm and applies to all panels.

We examined the expression of SOX2 protein in heterozygous and *Ascl1* knockout animals during embryonic development. SOX2 labels GBCs and Sus cells in the epithelium of hetereozygotes, as expected based on previous findings in adult OE [24] (Fig. 7). As described above, we used K18 as a marker of Sus cells. K18(–)/SOX2(+) cells are found below the neuronal layer among the basal cells. In the *Ascl1* knockout epithelium, SOX2 labels the vast majority of cells in the epithelium, which consist of both K18(+) Sus cells and K18(–) GBCs. The exceptions to this pan-epithelial SOX2(+) pattern are the duct cells present in the OE of embryos at E16.5 and beyond, which are SOX2(–) (data not shown).

**Figure 8:**
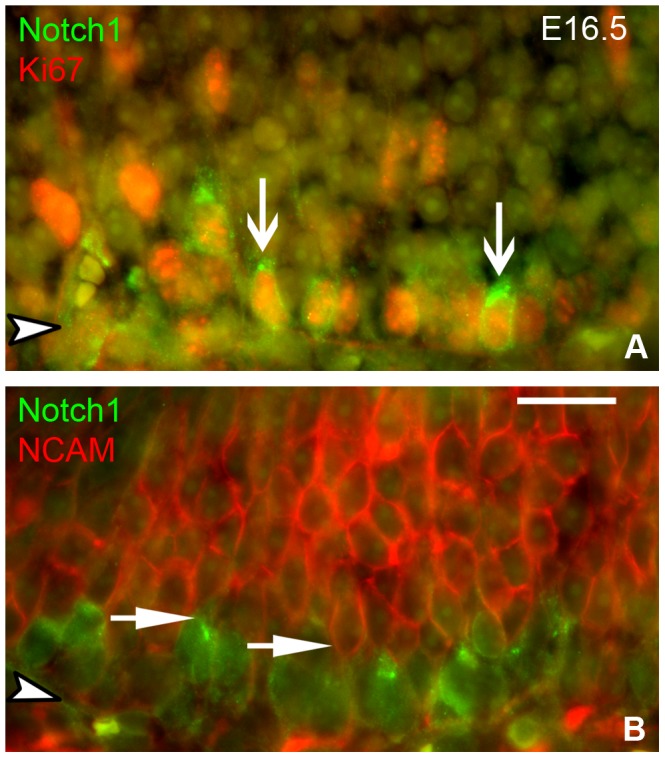
NOTCH1-labeled cells are proliferating and upstream of differentiated neurons. OE from **E16.5** wild type embryos was stained for **NOTCH1** and co-stained with **Ki67**, a marker of proliferating cells, and **NCAM**, a marker of neuronal differentiation. (**A**) Many **NOTCH1**(+) cells are **Ki67**(+) (vertical **arrows**). (**B**) The vast majority of **NOTCH1**(+) cells are **NCAM** (–). Horizontal **arrows** in **B** illustrate the border between **NOTCH1**(+) basal cells and the **NCAM** (+) neuronal layer. Arrowheads indicate basal lamina. Scale bar in **B** (20 µm) applies to both panels.

**Figure 9:**
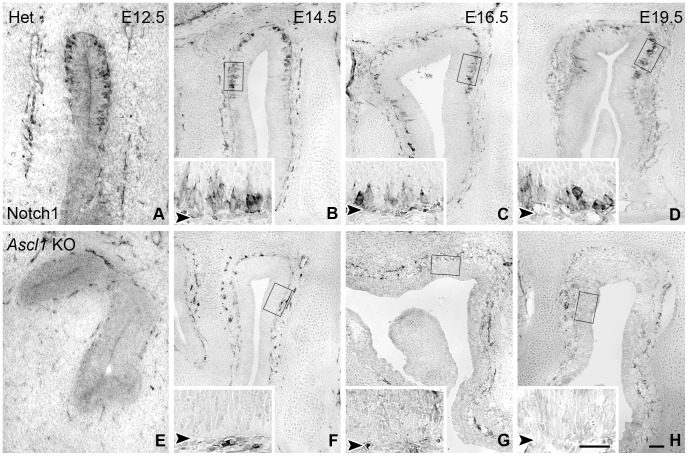
NOTCH1-labeled cells are absent from the *Ascl1* KO epithelium. OE harvested at various embryonic ages were stained for NOTCH1. Boxed regions are shown at higher magnification in the insets. Arrowheads in insets indicate basal lamina. (**A–D**) **NOTCH1** is expressed by basal cells throughout the developing OE of heterozygous mice; (**E–H**) Notch1 staining is largely absent from the epithelium of ***Ascl1*** **KO** mice. Higher magnification examination of the tissue on both sides of the basal lamina (insets **B–D**, **F–H**) demonstrate that **NOTCH1** staining in the ***Ascl1*** **KO** is found within the lamina propria, and not the epithelium, indicating that the absence of stained cell in the OE of knockout mice is not an artifact. Scale bar in lower magnification image of **H** (10 µm) applies to all panels. Scale bar for the high magnification inset in **H** is 25 µm and applies to all insets.

In the adult OE, NOTCH1 marks two populations of GBCs: ones that co-express SOX2, but not ASCL1 and are more “upstream”, and others that co-express NEUROG1, are likely to be producing neurons, and are “downstream” of ASCL1 (unpublished observations). Consistent with our prior unpublished observations in the adult, a population of basal cells in the embryonic OE of wild-type and heterozygous mice express NOTCH1, as shown by staining with two different anti-Notch1 antibodies (Fig. 8 and Fig. 9A–D). As expected, the vast majority of NOTCH1(+) cells are NCAM(–), since NCAM expression marks the onset of neuronal differentiation in the adult OE. Additionally many NOTCH1(+) basal cells are Ki67(+), a marker for mitotic cycling (Fig. 8). Both features are typical of GBCs.

Given the aberrant phenotype of Sus cells shown here and by others with respect to HES1 expression, and the usual role of HES1 as the downstream effector of the canonical Notch signaling pathway, we compared labeling for NOTCH1 in *Ascl1* knockout vs. heterozygous mice. In heterozygotes, NOTCH1(+) cells are seen amongst the basal cells at all stages examined – as early as E12.5 and extending into the early postnatal period **(** Fig. 9 **)**. In knockout littermates, anti-NOTCH1 labeling of the olfactory epithelium is eliminated at these same stages. In both heterozygous and knockout littermates anti-NOTCH1 stains cellular processes in the lamina propria to a roughly equal intensity at all time points examined, serving as a positive control for the absence of NOTCH1 reactivity in the knockout OE.

Because NOTCH1 is expressed by GBCs that express NEUROG1 and are downstream of Ascl1 in the adult, one might anticipate that the number of NOTCH1-expressing cells is less in the mutant OE, however their complete absence indicates that expression of NOTCH1 by more upstream GBCs – specifically the GBCs that normally co-express SOX2 and NOTCH1 but not ASCL1 [14] – is suppressed as a consequence of *Ascl1* knockout.

**Figure 10:**
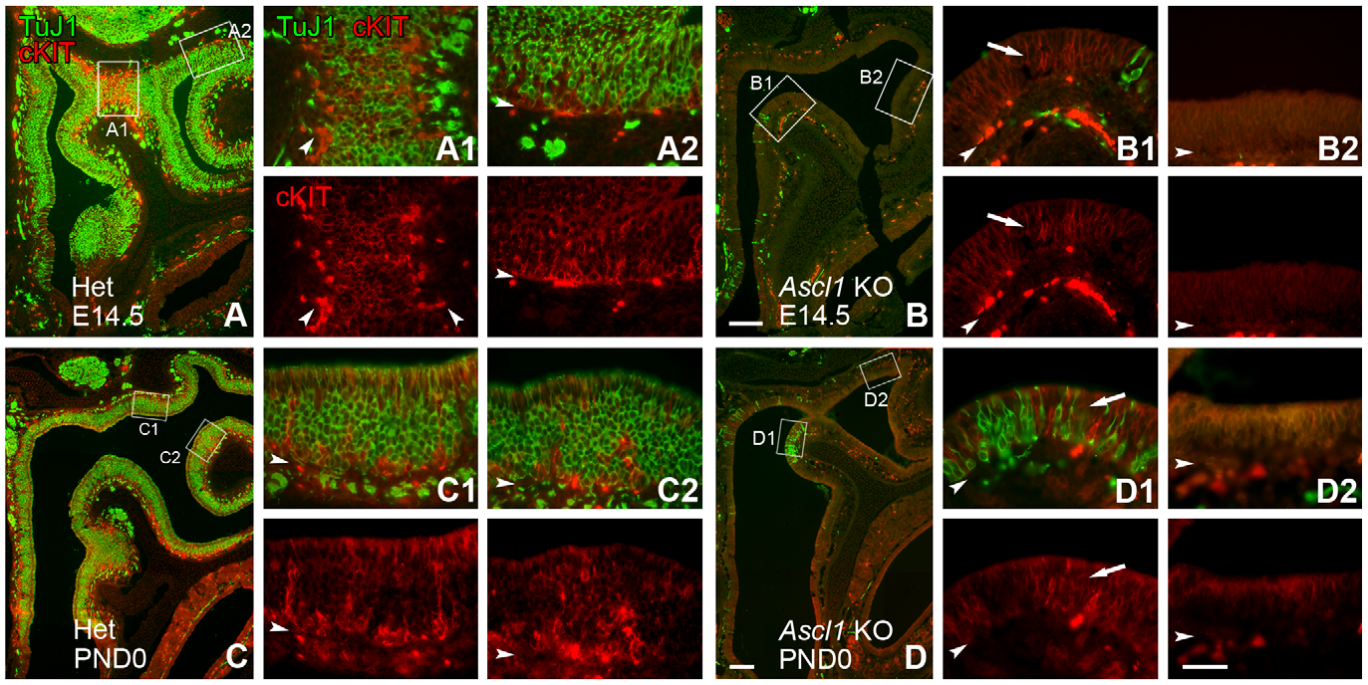
cKIT(+) basal cells are markedly reduced in *Ascl1* KO epithelium. Sections from **E14.5** and **PND0** ***Ascl1*** **HET** and **KO** animals were stained for **TuJ1** (which marks immature neurons heavily and also lightly stains some GBCs see Fig. 11 and Packard et al., 2011) and **cKIT**. (**A**–**D**) Low power images provide an overview of the distribution of **cKIT** relative to the neuronal population. Boxes are shown at higher magnification in panels to the right of each low magnification image (*upper* – merged red and green channels, *lower* – red channel). (**A, C**) In the **HET**, **cKIT**(+) basal cells are found deep to the neurotubulin (**TuJ1**) positive cells at both **E14.5** and **PND0**. Intensity of **cKIT** staining appears to increase from **E14.5** to **PND0**. (**B, D**) In contrast, much of the tangential extent of the ***Ascl1*** **KO** olfactory epithelium lacks detectable **cKIT** expression. The exception to this is found in domains of epithelium that are adjacent to or co-extensive with the areas that show neuronal sparing (**arrows** in **B_1_**, **D_1_**). **Arrowheads** mark the basal lamina. Scale bar in **B** is 100 µm and applies only to **B**. Scale bar in **D** is 100 µm and applies to **A**, **B**, and **D**. Scale bar in **D_2_** (*lower*) is 50 µm and applies to all high magnification panels.

**Figure 11:**
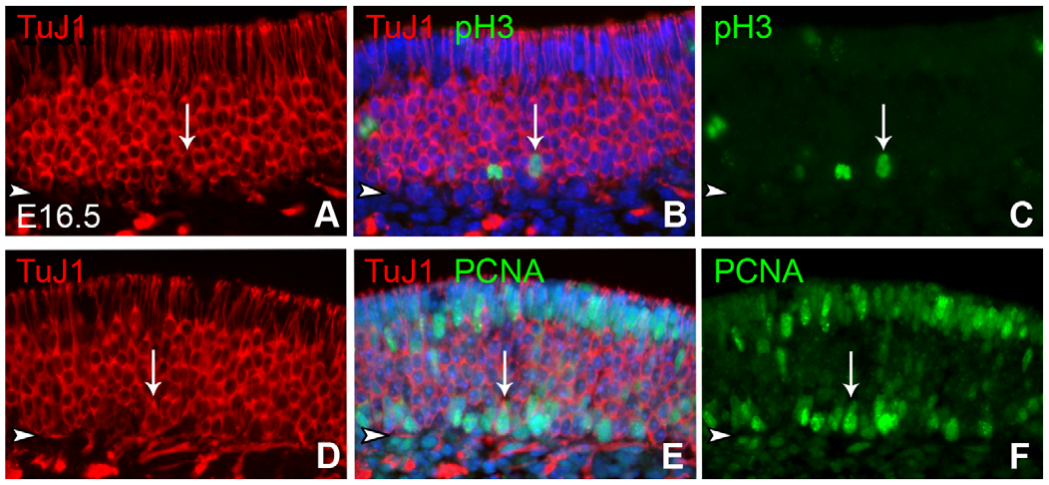
A subset of TuJ1(+) cells are proliferating. Sections of OE harvested from E16.5 wild type animals were stained with **TuJ1** and co-stained with phospho-histone 3 (**pH3**, **A–C**) or proliferating cell nuclear antigen (**PCNA**, **D–F**) and Hoechst 33258 to label nuclei. (**A, D**) Red channel only. (**B, E**) Merged images including Hoechst nuclear stain (blue). (**C, F**) Green channel only. **Arrows** indicate double positive cells. Scale bar in **F** is 25 µm and applies to all panels.

The expression of c-KIT also marks a subset of GBCs in the adult OE, but the receptor is absent from those GBCs that are functioning as multipotent progenitors at the earliest stage in the regeneration of the adult rat OE after injury (Schwob, unpublished observations). In addition, expression of *Steel* (a.k.a stem cell factor, SCF or kit-ligand) is markedly increased in the Sus cells of *Ascl1* knockout mice [15]. Accordingly, we assayed for expression of c-KIT in olfactory epithelium of heterozygous and *Ascl1* knockout animals. At E14.5, c-KIT expression in the OE of *Ascl1* heterozygotes is limited mainly to basal cells that lie just deep to the band of TuJ1(+) cells (Fig. 10). In the embryonic and adult mouse OE, intense staining with TuJ1 marks immature olfactory neurons [32]. But it also stains some proliferating GBCs that are identified as such by their co-labeling with proliferating cell nuclear antigen (PCNA) or phosphorylated Histone 3 (pH3) (Fig. 11 and [32]). Based on co-labeling with the bHLH transcription factor NEUROG1, the TuJ1(+) GBCs are classified as an immediate neuronal precursor stage far along the path toward neuronal differentiation [32]. Because c-KIT does not overlap with Tuj-1, c-KIT expression in the wild-type or heterozygous embryonic OE looks to be characteristic of GBCs that are slightly upstream of the terminal mitotic stage of neurogenesis.

Throughout embryonic development into the immediate postnatal period, the c-KIT(+) GBCs extend throughout the anterior-posterior and medial-lateral extent of the olfactory epithelial sheet (Fig. 10). From E14.5 to PND0, their numbers increase (Fig. 10). In contrast to their frequency in, and breadth across, the heterozygote OE, the number and distribution of c-KIT(+) basal cells is markedly abnormal in the knockout OE, but the differences are complex. Overall, there are fewer c-KIT(+) basal cells in the knockout. Moreover, their distribution is much less even, *i.e.*, much more focal, in the knockout OE. Swaths of the epithelium of the *Ascl1* knockout lack c-KIT(+) cells at E14.5 and PND0 (Fig. 10B_2_, D_2_) but there are also foci in which c-KIT (+) cells form clusters (Fig. 10B_1_, D_1_). These foci are consistently co-extensive with the scattered groupings of TuJ1(+) neurons (identified by their elaboration of axons and dendrites) that develop despite the null mutation of *Ascl1*. For example, the co-clusters of c-KIT and TuJ1 are seen in the part of the OE lining the dorsal recess and at points near the fusion of endoturbinates with the cribriform plate, but not elsewhere (cf. Fig. 10B_1_, D_1_ vs. B_2_, D_2_, respectively). Conversely, areas that lack TuJ1(+) neurons also lack c-KIT(+) basal cells. Thus, c-KIT(+) GBCs are largely absent from the epithelium of the knockout, but where present may contribute to the generation of those neurons that form despite the null mutation of *Ascl1*.

## Discussion

The results presented here provide novel insights into the regulation of olfactory epithelial assembly during embryonic development by analyzing the development and differentiation of the other, non-neuronal cell types of the *Ascl1* knockout olfactory epithelium. Sus cells in the knockout epithelium develop on time and express virtually all of the same markers as do Sus cells in the wild-type with the exception that they express lower levels of HES1, perhaps because HES1’s role in suppressing its canonical target *Ascl1* has been obviated by the null mutation of that gene. The production of horizontal basal cells (HBCs) is aberrant but not aborted in the olfactory epithelium of *Ascl1* knockout mice; as the population is still incomplete in age-matched littermates when the mutant pups die or are killed perinatally, we cannot know whether or not HBC number might eventually catch-up in the knockout mice. In contrast, duct/gland development occurs normally with respect to timing and number of duct/gland units.

Several abnormalities of the GBC population are also prominent. First, as a result of *Ascl1* knockout, NOTCH1, which is normally expressed by basal cells, is no longer demonstrable in the OE, and HES1 is markedly lower overall. The absence of NOTCH1 expression can be attributed in part to the failure to form the NOTCH1/NEUROD1/NEUROG1-expressing GBCs in the knockout OE that are usually downstream of Ascl1 and act as immediate neuronal precursors in the normal epithelium (Krolewski and Schwob, unpublished results). However, NOTCH1 has also become undetectable on the GBC precursors that are upstream of *Ascl1* that normally also express the receptor [14]. The absence of detectable NOTCH1 on upstream GBCs indicates reciprocity between NOTCH1 and its downstream effector HES1 on the one hand, and the downstream target *Ascl1* on the other [33], [34], which is consistent with the canonical relationship whereby NOTCH1 activation of *Hes1* is followed by HES1 suppression of *Ascl1* in other tissues [35]. These data, too, indicate that high levels of HES1 are not required for the making of Sus cells from the multipotent progenitors of the OE in the setting of the knockout. (By extrapolation, the upregulation of Hes1 mRNA and protein in the GBCs of the MeBr-lesioned adult OE that are destined to make Sus cells may be serving to suppress *Ascl1* and neurogenesis, rather than stimulating Sus cell formation directly [13], [23].) Second, there is an expansion of the population of upstream Sox2(+) GBCs in apparent reaction to the elimination of Ascl1(+) transit amplifying GBCs. Finally, C-KIT expression appears to be largely downstream of ASCL1, but apparently can also be activated in an *Ascl1*-independent manner in association with the extremely limited capacity of the knockout epithelium to generate olfactory neurons.

The identification of the various cell types found within the olfactory epithelium depends upon immunophenotyping and morphologic characterization. The reagents used for detection of the differentiated cell types of the OE – including TuJ1, PGP9.5, K18, and K14– are widely used for studying both neuronal and epithelial tissues [1], [5], . For a number of these differentiated cell antigens, multiple antibodies raised in various species result in the same staining pattern [1], [19] and multiple antibodies raised against different epitopes result in the same labeling pattern (e.g., the equivalence of the different NOTCH1 antibodies described here). The extensive use of these reagents, as well as their cross-species validation in mouse, rat, and human increases the confidence with which we can interpret the molecular characterization of the cell types labeled by the antibodies.

### Implications of Altered Epithelial Composition in the Ascl1 Knockout Mice

The results reported here offer insight into how different cell types are produced during the ongoing stress of persistent neurogenic failure caused by *Ascl1* null mutation. Particularly striking is the abnormal pattern of HBC production when neurogenesis is aborted, in contrast to duct/gland and Sus cells, which are generated at the usual time and to the usual extent, even though some aspects of their differentiation may be altered. A close examination of the differentiation of HBCs during development is instructive. Other work from the lab has shown that a minority of p63(+)/K14(−) GBC-like cells – p63 being a transcription factor that is required for HBC differentiation and a marker for candidate HBC precursors in the embryonic and perinatal OE – normally express ASCL1 very early in the embryonic emergence of the HBCs [31]. However, a much larger number of p63(+) cells express HES1, and not ASCL1, and for a longer period of time, suggesting that the delay observed here in the *Ascl1* mutant mice is not a direct effect of gene knockout and, rather, that the down-regulation of NOTCH1 and HES1 in the *Ascl1* knockout epithelium is responsible for that delay. Moreover, the near-normal production of HBCs in the region adjacent to the RE also demonstrates that there is no absolute block to their genesis, as does the emergence of rare HBCs in parts of the OE at a distance from that boundary. The differential between the relatively abundant population of HBCs in the OE-RE boundary zone and the more pronounced deficit elsewhere does suggest that there are different mechanisms responsible for the emergence of HBCs in the two territories, at least in the knockout. Given the confluent monolayer of K5/14(+) basal cells in the RE, one should consider the hypothesis that HBCs in the part of the OE near the OE-RE boundary have migrated in from the RE.

**Figure 12:**
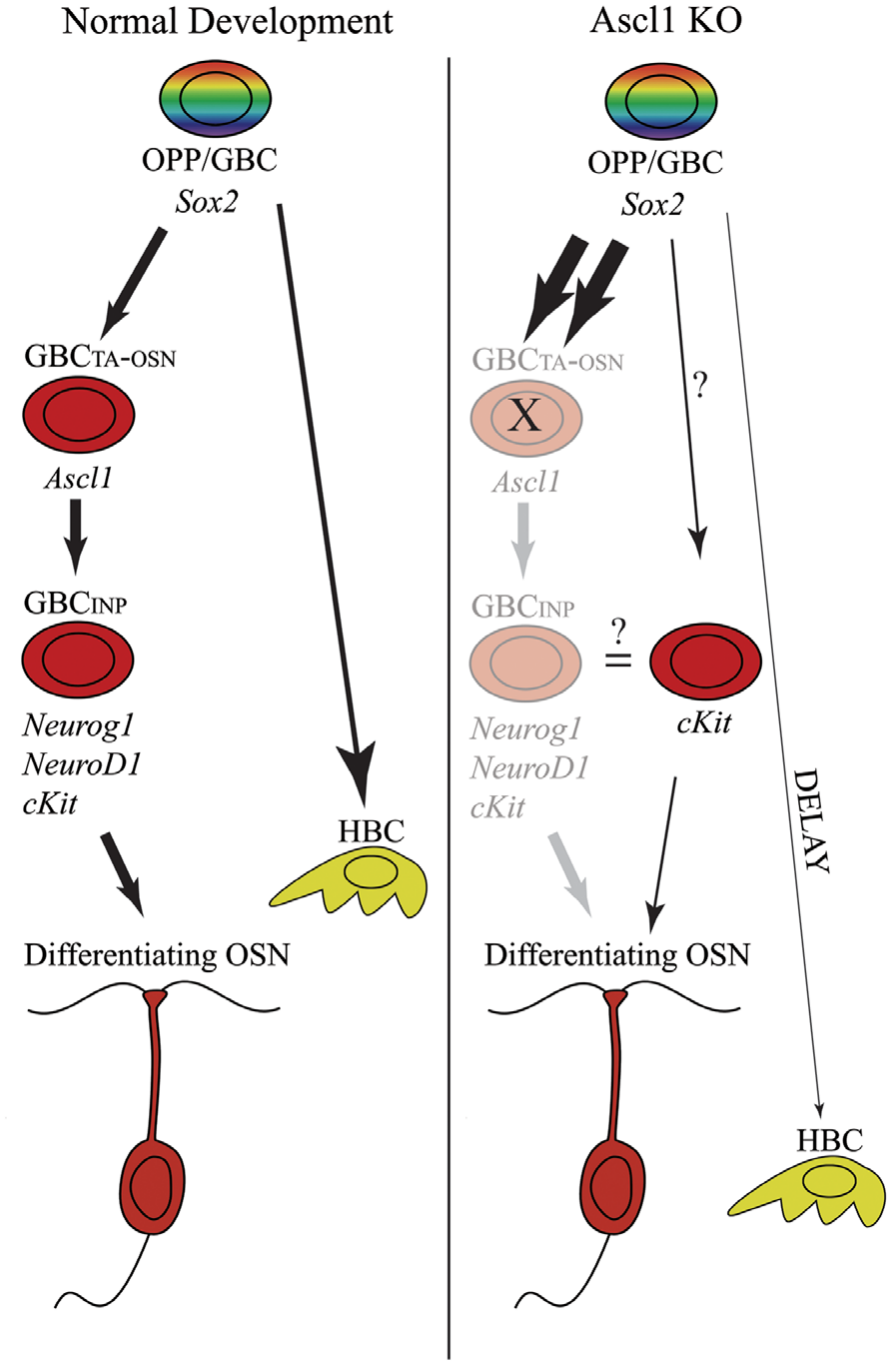
A model of how cell differentiation of non-neuronal cells is affected by the neurogenic failure in *Ascl1* knockout mice. The model depicts proposed cell lineage relationships during **normal development** in the embryo (**left side**) as compared to *Ascl1* knockout (**Ascl1 KO**) animals (**right side**). Italics designate genes expressed by the various cell types. Arrow thickness on the *left* and *right* sides of the model represent differences in differentiation pathways utilized in normal development as compared to the *Ascl1* knockout – **thicker arrows** indicate a pathway that is used more, while **thinner arrows** indicate less. **Faded cell types** on the knockout panel (**right**) symbolize the loss of those cell stages because of *Ascl1* knockout (Cau et al., 1997). *Ascl1* knockout pushes the epithelium toward the expansion of upstream OPP/GBCs and away from HBCs (which are normally generated by OPP/GBCs; Packard et al., 2011) in the face of aborted production of neurons. However, to a limited degree, neurons are made by, or in apparent association with, cKIT-expressing progenitors. Abbreviations: OPP/GBC = olfactory placodal progenitor/globose basal cell, GBC_TA-OSN_ = transit amplifying globose basal cell producing olfactory sensory neurons, GBC_INP_ = immediate neuronal precursor, HBC = horizontal basal cell.

Thus, the deviations from the normal course of HBC development suggest a more tightly integrated and interdependent epitheliopoeitic process than had been previously appreciated [2], [3], [9]. By this formulation, the emergence of HBCs is delayed in the *Ascl1* knockout because the multipotent progenitors of the embryonic epithelium are diverted away from the production of this reserve population of multipotent cells in an apparent attempt to compensate for neurogenic failure (Fig. 12).

There are at least two possible explanations for this observation: (1) HBCs, being a reserve population, are not set aside because the absence of neurons is sensed (as is true when neurons die in the adult epithelium, causing accelerated basal cell proliferation and neuron production, as a consequence), which diverts the reserve-generating progenitors toward attempting, but failing, to make neurons. Here again, down-regulation of Notch1-Hes1 signaling is likely to favor the formation of neurons over non-neuronal cells, which we have shown to be the consequence of blocking Notch signaling in the adult OE (Schwob, unpublished observations) and by analogy to other neural systems [38]. Moreover, Notch1 is often tightly regulated by the status of progenitor cells and their progeny. For example, when B lymphocyte differentiation is blocked by *Pax5* null mutation, the pro-B cells up-regulate Notch1, although the mechanism(s) underlying the feedback loop in the case of the immune system is (are) not yet understood [39]. (2) The relative block to HBC differentiation delay is a side-effect of a larger phenomenon of altered tissue homeostasis, rather than a direct consequence.

The minimization of the Notch1-Hes1 axis tends to favor the former explanation, as does the relatively inconsequential change in the other populations. In either case, the flow into the HBC reserve population is highly regulated (and is mirrored by the complexities of HBC activation to multipotency in the adult epithelium [31]). The results suggest that multipotent progenitors can be directed away from or toward a specific cell type as needed, i.e., away from HBCs and toward upstream (i.e., Sox2-expressing) but NOTCH-negative GBCs depending on the cell types present and active at a given time. This diversion towards and away from different lineages appears distinct from the consequences of ablation of specific cell types in other sensory systems (such as pigmented epithelium in the retina or hair cells of the inner ear), which tends to result in increased size of other populations [40] or transdifferentiation to replace the ablated population [41], rather than the apparent delay in differentiation of an accessory cell type as we report here.

If down-regulation of the Notch1-Hes1 axis is the effector, what might be the signal(s) communicating the need for suppressing the cascade and thereby favoring neurogenesis? BMP4, which is produced by OSNs, is an attractive candidate for the factor that provides feedback regarding the status of the neuronal population and can down-regulate proliferation and neurogenesis [42]. For example, BMP4 drives progenitor cells to degrade ASCL1 via ubiquitin-mediated proteolysis [43]. Moreover, Notch and BMP signaling interface in other tissues. For example, BMP4 is upstream of, and maintains, NOTCH1 expression in the hair follicle [44], leaving open the possibility that the loss of BMP4 from neurons in the OE of *Ascl11* knockout mice influences NOTCH1 expression in an analogous manner. In addition, the expression of *Ascl1* is up-regulated in response to *Foxg1*-driven, Cre-mediated deletion of *Notch2* [45], suggesting that NOTCH2 expression in Sus cells may also be a component of the feedback loop. Left unexplained is the residual, albeit reduced, expression of HES1 despite the elimination of NOTCH1. NOTCH2 expression in Sus cells may again be responsible. However, in other settings, HES1 can respond to other pathways: for example *Hes1* has been shown to be activated by the Hedgehog pathway, independent of NOTCH1 activation in MNS-70 cells, a neural stem cell line [46].

In sum, the aberrancies in HBC differentiation and the down-regulation of NOTCH1 signaling in GBCs may be tied together and reflect the pathways by which the existing population of neurons is known to influence neurogenesis.

The other abnormality within the GBC population is the altered expression of c-KIT and its apparent association with those neurons that form despite the elimination of *Ascl1*. The results suggest that c-KIT is dependent on, or downstream of, ASCL1 and may play a crucial role in the late stages of neurogenesis. Evidence for this includes the spatial coincidence of ASCL1-independent neuronal production with c-KIT expression (this work) as well as enhanced expression of kit-ligand (stem cell factor, SCF/Steel) by Sus cells when neuron production is globally disrupted by *Ascl1* mutation [15]. While the occasional production of neurons in the *Ascl1* knockout has been described before [11], the patchy distribution and comingling of c-KIT(+) progenitors and TuJ1(+) cells across the epithelial sheet suggest that the neurogenic pathways converge again to use conserved regulatory machinery, i.e., c-KIT, in these breakthrough areas (Fig. 12). This alternate pathway may also account for the *Lhx2*- and *Ebf1*-expressing cells previously described in the OE of *Ascl1* mutants [11]. By analogy with the hematopoietic system, *Lhx2* and EBF1 expression have both been localized to c-KIT (+) progenitors, and c-KIT signaling is necessary for LHX2-stimulation of colony forming activity [47], [48], thereby setting a precedent for interaction and co-expression of these genes.

The current data provide a novel insight into the rich web of signaling interactions that try to react to and compensate for specific stresses and demands on the developing olfactory epithelium. It reveals additional epistatic relationships among proteins and cell types affected by *Ascl1* knockout and the ongoing failure of the neuronal lineage that ensues.
